# What Is the Subjective Cost of Cognitive Effort? Load, Trait, and Aging Effects Revealed by Economic Preference

**DOI:** 10.1371/journal.pone.0068210

**Published:** 2013-07-22

**Authors:** Andrew Westbrook, Daria Kester, Todd S. Braver

**Affiliations:** Department of Psychology, Washington University in Saint Louis, Saint Louis, Missouri, United States of America; Inserm, France

## Abstract

It has long been assumed that people treat cognitive effort as costly, but also that such effort costs may vary greatly across individuals. Individual differences in subjective effort could present a major and pervasive confound in behavioral and neuroscience assessments, by conflating cognitive ability with cognitive motivation. Self-report cognitive effort scales have been developed, but objective measures are lacking. In this study, we use the behavioral economic approach of revealed preferences to quantify subjective effort. Specifically, we adapted a well-established discounting paradigm to measure the extent to which cognitive effort causes participants to discount monetary rewards. The resulting metrics are sensitive to both within-individual factors, including objective load and reward amount, and between-individual factors, including age and trait cognitive engagement. We further validate cognitive effort discounting by benchmarking it against well-established measures of delay discounting. The results highlight the promise and utility of behavioral economic tools for assessing trait and state influences on cognitive motivation.

## Introduction

Is cognitive effort costly? There is a long tradition within psychology of characterizing humans as “cognitive misers” [1], who conserve cognitive effort, all else being equal ([2], [3], also [4] on general effort avoidance). This implies that individuals value their effort, treating it as costly. Yet, the value of cognitive effort is likely to be highly subjective, varying across individuals and populations [3], [5], [6]. Meaningful individual and group differences in the valuation of cognitive effort would have broad implications. Willingness to expend effort is rarely controlled in cognitive and neuroscience research, and therefore constitutes a pervasive confound. Specifically, variability in behavioral and neural measurements during task performance may reflect cognitive motivation as well as cognitive ability. This may be particularly relevant for understanding apparent cognitive deficits observed in clinical populations featuring anergia or avolition [7]–[10], or among older adults [11], [12].

The broad implications of this question have motivated self-report scales that provide both state markers of cognitive effort, such as the NASA Task Load Index (NTLX) [13] and trait measures of effortful task engagement, like the Need for Cognition Scale (NCS) [14]. Yet, the well-known difficulties of self-report point to the need for objective measures. Initial efforts in this regard have examined behavioral biases to avoid cognitive effort in free-choice tasks, yielding promising results [3], [5], [6]. Even greater traction may be gained, however, by adapting behavioral economic tools such as revealed preference and subjective value. Revealed preference procedures, in which preferences are inferred from choice behavior rather self-report, can be used to provide quantitative measures of the subjective and economic value associated with different choices. Discounting is one such formalism, in which the value of a cost factor is measured by the extent to which it reduces preference for a given reward. Discounting paradigms have been productively applied in behavioral- and neuro-economics to study diverse costs, from delay and risk [15]–[18], to physical effort [19], [20], and may prove useful for measuring subjective cognitive effort as well [3], [21], [22].

Here we introduce a novel discounting paradigm that provides an objective measure of the cost of cognitive effort. We investigate the validity and utility of this measure by: 1) establishing a parametric relationship with objective cognitive load; 2) identifying meaningful individual differences in effort costs; 3) demonstrating that older adults (OA) find effort more costly than younger adults (YA); and 4) benchmarking effort discounting, in terms of sensitivity to experimental factors and age differences, against the well-established domain of delay discounting.

### The Cognitive Effort Discounting Paradigm (COG-ED)

**Figure 1:**
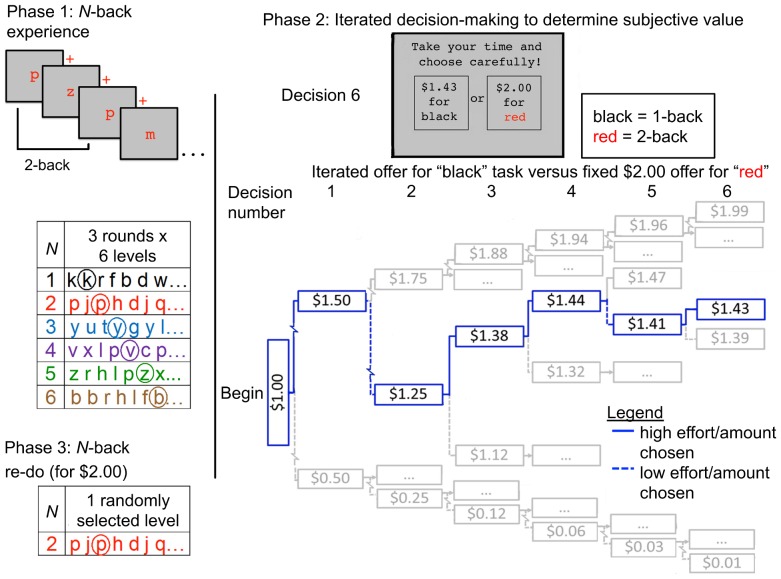
Cognitive effort discounting (COG-ED) paradigm. Task structure including *N*-back practice, effort discounting, and *N*-back re-do.

The key feature of this paradigm is that participants choose whether to perform a low-effort task for a small monetary reward or a high-effort task for a larger reward (Figure 1). Multiple choices are made, and the amount offered for the low-effort task is titrated until subjective equivalence is reached (the offers are equally preferred). The additional amount required to make the high- and low-effort task equivalent quantifies the cost of cognitive effort, or the degree to which increased cognitive effort diminishes the value of task engagement. The objective load of the high-effort task can also be varied parametrically. As such, the procedure is formally analogous to the estimation of discounting across a range of delays in delay discounting. Unlike discounting procedures involving hypothetical costs or rewards, participants make choices about tasks that they are actually paid for re-doing, promoting test validity.

Here, we vary objective cognitive load using the *N*-back. The *N*-back has a number of attractive properties: a) it is a well-established probe of working memory and cognitive control [23], b) it is phenomenally effortful [24], and c) load can be varied parametrically by increasing *N* [25]. Also, time-on-task can be fixed across load levels. Participants are given extensive *N*-back practice prior to decision-making, in order stabilize performance and familiarize participants with the subjective experience of each load level.

## Methods and Materials

### Experiment 1 Participants

Twenty-five younger adult participants were recruited (YA; ages 18–30, Mean age: 21.8) from the Washington University community, and 30 older adults were recruited from a volunteer older adult (OA) subject pool. Participants were native English speakers, neurologically normal with no history of mental disorder or drug abuse, and not currently taking any psychotropic medication. The Six-Item Cognitive Impairment test [26] was administered to screen for dementia among OA. Five OA participants that passed screening did not complete the protocol or were excluded for failure to comply with instructions, leaving a sample of 25 OA participants for study (ages 63–88, Mean age: 75.1). Participants were paid $10/hour for their participation plus payment for levels re-done, selected at random from among all discounting choices [27]. Procedures were approved by the Washington University in Saint Louis IRB. All participants provided informed, written consent.

### Experiment 1 Procedure

Each session began with *N*-back experience, which was followed by the effort discounting paradigm (Figure 1). Finally, participants completed additional *N*-back rounds based on a random selection from among their choices in the discounting procedure. Additionally, a number of self-report measures were administered. The NTLX was completed after each level of *N*-back experience to assess mental, physical, and temporal demand, effort, performance, and frustration. Immediately prior to decision-making, participants completed an additional self-report questionnaire reflecting on their task experience. Participants also completed the NCS, a trait index of the extent to which individuals engage with and enjoy cognitively demanding activities.

The practice phase with the *N*-back included three runs for every load level, each comprised of 64 items (consonants, 24-point Courier New font, 16 targets, in colors uniquely identifying levels, *N*). Participants had 1.5 s to respond to each item by button press, after which items were replaced by fixation cross. The inter-item interval was 3.5 s. Lures (items within *N* +2, but not exactly *N*, positions after last presentation) were included in *N*-back stimulus lists to increase level difficulty: eight for *N* = 1, six for 2, five for 3, and three for *N* = 4, 5, and 6. Participants were given feedback about run-wise “% of targets” and “% of non-targets correct”. To motivate engagement, and to prevent participants from responding, e.g., “Non-target” at the expense of the “Target” score, participants were also given feedback of “Good job!” if both scores were above 50% or “Please try harder!” otherwise.

In the discounting procedure, participants made choices between repeating a higher level for more money or a lower level for less money. Analogous to adjusting-immediate-amount (AIA) procedures [28], choices were always between a smaller, variable reward (starting at $1.00) for the easiest level (*N* = 1) and a larger, fixed reward ($2.00) for each of the harder levels (*N*>1). Levels were referred to by identifying color, to avoid anchoring effects [29]. If, on a given trial, the larger offer was selected, the offer for the easiest level was increased, and if the smaller offer was selected, it was decreased. Each time a choice was made, adjustments were half as much as on the prior adjustment. Participants made six choices so that adjustments following their final choice for each level were $0.015, and the resulting amount was taken as the participant's point of indifference. YA made 5 levels (*N* = 2–6) * 6 offers = 30 choices and OA made 3 levels (*N* = 2–4) * 6 offers = 18 choices. Offer order was randomized and non-nested [28]. Participants were instructed that one of their choices would be randomly selected for them to repeat up to 10 more times, receiving payment for each repetition. They were further instructed that payment was contingent on “maintaining their effort”, but not on performance, and that “behavioral clues” would be used to monitor effort. All participants completed their randomly selected choice exactly four more times and were paid the associated amount for each repetition. Aside from the pencil-and-paper self-report measures, the entire protocol was programmed and administered in E-prime 2.0 (Psychology Software Tools, Inc., Sharpsburg, PA).

A second experiment was conducted to benchmark COG-ED against delay discounting, a well-established domain that also has been shown to index meaningful individual and age-group differences [30], [31]. In Experiment 2, a new sample of participants was recruited to perform both delay and effort discounting tasks in the same experimental session.

### Experiment 2 Participants

An additional 17 YA (ages 18–24, Mean age: 20.5) and 16 OA (ages 67–89, Mean age: 78.0) were recruited from Washington University and the OA subject pool, respectively. Years of education were reliably higher for OA than YA (OA range 12–18, Mean years: 16.4; YA range 13–18, Mean years: 14.9; Wilcoxon rank sum, *P*<0.01).

### Experiment 2 Procedure

Participants completed both delay discounting and COG-ED paradigms within a single session. We used the same procedure as in Experiment 1, except for the following changes. First, both age groups completed three runs of practice with *N*-back levels = 1–4. Second, participants were offered two base amounts ($1 and $5) for the highest level, rather than just one ($2). Participants thus made 3 levels (*n* = 2–4) * 6 offers * 2 amounts = 36 effort discounting choices. In delay discounting, participants made choices regarding hypothetical rewards and six delays: one week, six months, and 1, 3, 5, and 10 years. Two rewards were used, $1,000 and $25,000, to examine amount effects. Participants thus made 6 delays * 6 offers * 2 amounts = 72 delay discounting choices. As before, offer order was randomized and non-nested. To gain greater insight into the factors that influenced decision-making in COG-ED, we also asked participants to rate the degree (Likert scale, 1–10) to which their decisions were based on offer amounts, effort, desire to do well, desire to challenge themselves, etc. Finally, we collected income (family household for students, average of highest three pre-retirement years, for retirees), indicated by one of 9 ranked bins (<$20K, $20–40K,…, $120–150K, or >$150K), to test for effects on marginal utility of our offers.

## Results

### Experiment 1

#### Parametric effects of N-back load on performance and self-report indicators of effort

We first tested for load effects on performance (Table 1). Consistent with prior work (e.g., [25]), linear contrast ANOVAs revealed effects of Level (*N*) on Performance (signal detection *d'*) for YA (*N* = 2–6, *F*_1,122_ = 92, *P*<0.01, η^2^ = .43) and OA (*N* = 2–4, *F*_1,72_ = 29, *P*<0.01, η^2^ = .29), validating the load manipulation. There was also a linear effect of increasing Objective Load associated with slower response times among YA (*F*_1,122_ = 5.10, *P* = 0.03, η^2^ = 0.04), but not OA (*F*_1,72_ = 0.55, *P* = 0.46).

**Table 1:** *N*-back response times (correct trials), *d'* (all trials) for YA and OA.

	*Young Adults (n = 25)*				*Old Adults (n = 25)*			
	*RT (msec)*		*d'*		*RT (msec)*		*d'*	
*Load Level (N)*	*M*	*SD*	*M*	*SD*	*M*	*SD*	*M*	*SD*
*1*	592	101	3.42	0.97	785	110	3.13	0.81
*2*	714	136	2.84	0.72	886	128	2.11	0.53
*3*	734	146	2.26	0.73	884	124	1.74	0.40
*4*	713	126	1.65	0.64	859	130	1.39	0.48
*5*	687	119	1.53	0.77	-	-	-	-
*6*	640	151	1.08	0.63	-	-	-	-

Self-reported task perceptions also support the presence of load-related increases in subjective effort (Table 2). Linear contrasts of NASA Task Load Index (NTLX) scores by *N*-back load level (*N* = 1–4), indicated that participants perceived greater mental and physical demands, increasing rush, increasing failure, greater demands for effort, and increasing frustration with increasing *N* (all *P*'s<0.01).

**Table 2:** Self-report NASA Task Load Index Scores (scale: 0–21) by level, *N*.

	*Young Adults (N = 25)*					
	*Mental Demand*	*Physical Demand*	*Temporal Demand*	*Failure*	*Effort*	*Frustration*
*N*	*M (SD)*	*M (SD)*	*M (SD)*	*M (SD)*	*M (SD)*	*M (SD)*
1	6.3 (3.8)	2.6 (1.5)	8.3 (5.2)	6.3 (4.1)	6.7 (3.4)	5.9 (4.9)
2	11.9 (3.5)	3.7 (3.0)	9.6 (4.5)	7.4 (3.5)	11.1 (3.7)	7.9 (4.9)
3	15.2 (3.3)	5.0 (4.1)	11.7 (4.4)	10.3 (4.0)	14.3 (3.1)	10.3 (5.2)
4	16.4 (3.3)	5.3 (5.0)	13.1 (4.5)	13.0 (3.9)	15.5 (3.2)	11.7 (5.4)
5	17.0 (2.8)	5.8 (5.7)	13.4 (5.6)	13.4 (4.2)	15.7 (3.8)	11.9 (5.1)
6	18.4 (2.0)	6.3 (6.3)	13.4 (6.0)	15.2 (4.4)	15.6 (4.9)	10.8 (6.2)
	*Older Adults (N = 25)*					
1	9.6 (5.4)	4.1 (3.5)	8.2 (5.4)	7.6 (4.4)	9.0 (5.7)	5.3 (4.5)
2	13.0 (5.4)	6.4 (5.2)	11.5 (5.0)	10.6 (4.2)	13.3 (5.5)	8.3 (5.0)
3	14.2 (5.6)	7.6 (5.2)	12.4 (5.2)	12.0 (4.5)	14.3 (5.6)	10.0 (5.9)
4	15.7 (5.4)	8.5 (6.0)	13.3 (5.5)	12.5 (5.1)	14.8 (5.2)	11.0 (6.3)

#### Parametric effects of task load on effort discounting

**Figure 2:**
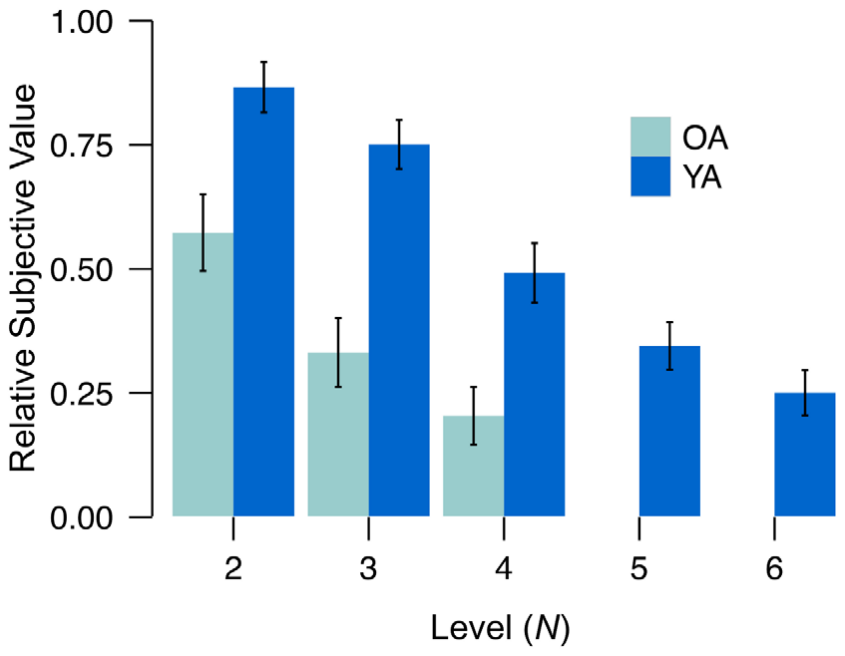
Subjective value of rewards for task engagement across multiple levels of the *N*-back for OA and YA. Subjective value decreases linearly with load (*N*). OA discount more than YA at all levels (*N* = 2–4). SE are shown. *n_YA_* = 25 participants * 6 levels = 150 and *n_OA_* = 25 participant * 4 levels = 100. Data also included for illustrative purposes in [51].

The key analysis was to determine whether participants showed reduced subjective value of offers requiring greater effort. Results clearly show greater discounting of more demanding *N*-back levels (Figure 2). Reliable linear contrasts obtained for both YA (*N* = 2–6, *F*_1,122_ = 103, *P*<0.01, η^2^ = 0.46) and OA (*N* = 2–4, *F*_1,72_ = 14, *P*<0.01, η^2^ = 0.17), indicate that effort costs increased with objective load. For example, the mean, relative subjective value (SV) of a $2.00 offer for redoing *N* = 4, was .49 * $2.00 = $0.98 for YA and .20 * $2.00 = $0.40 for OA. Thus, YA required an additional $1.02 of payment to perform the 4-back instead of the 1-back, whereas OA required an additional $1.60 (conversely, YA would forego $1.02 to avoid the 4-back and perform the 1-back instead, while OA would forego an additional $1.60).

An important question is whether decreasing SV with increasing load can be fully explained by declining performance. We examined this issue in multilevel models controlling for load-related performance (*d'* and mean response time, mRT) using subject-specific intercepts and separate models for YA and OA. Multilevel models are useful because they allow for pooling of information across participants to more accurately estimate within-subject effects of load and performance across relatively few observations (four for each participant: *N* = 1–4) while accounting for between-subject variability. In our model, between-subject variability is modeled with subject-specific intercepts *B*_0*j*[*i*]_. Following [32], subscripts refer to participant *j*, the (within-participant) load-level *i*, and *j*[*i*] indicates the nesting of participant-level *i* within participant *j*. Load level, *N*, is further subscripted to indicate that loads were recoded from *N* = −1.5 to *N* = 1.5, centering at *N* = 2.5. Subject-specific intercepts are given a normal distribution with mean and variance terms in the second level of the model. Multilevel models were fit using the *nlme* package, version 3.1-109, in R (http://www.r-project.org/); parameters were estimated by maximizing restricted log-likelihood [33].
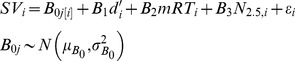
(1)


In both age groups, load was a reliable predictor of SV, even controlling for performance (Table 3). Thus, though errors and time-on-task increased with task load, parametric load effects on discounting were independent of these task performance changes.

**Table 3:** Multilevel models of subjective value, as predicted by performance (d'), mean response time (mRT, seconds), and *N*-back level.

Term	*B*	*SE*	*df*	*t*	*p*
Young Adults (n = 4 levels * 25 participants = 100)					
*d'*	0.05	0.03	72	1.70	0.09
*mRT*	−0.05	0.19	72	−0.24	0.81
*N_2.5_*	−0.13	0.02	72	−4.98	<0.01
Older Adults (n = 4 levels * 25 participants = 100)					
*d'*	0.08	0.05	72	1.49	0.14
*mRT*	−0.56	0.26	72	−2.16	0.03
*N_2.5_*	−0.21	0.03	72	−5.95	<0.01

We note that we used SV at *N* = 1 (SV_1_) for estimating our multilevel models despite the fact that SV_1_ was given an assumed theoretical value (SV_1_ = 1), rather than experimentally determined. We included this theoretical data point since the intent of the multilevel modeling was to evaluate the effects of performance and load on the decline in SV relative to this reference value (SV_1_). However, to ensure that our conclusions were not unduly biased by inclusion of this theoretical data point, we also estimated multilevel models of SV data restricted to *N* = 2–4 (centered at *N* = 3). To retain information about the drop in performance measures (mRT and *d*') relative to their values at *N* = 1, we used the ratio of performance values at each level to their values at *N* = 1 as predictors. Removing SV_1_ made no difference in the key results, as the main effect of load on SV was still present for both YA (*B* = −0.17, *SE* = 0.03, *P*<0.01) and OA (*B* = −0.16, *SE* = 0.04, *P*<0.01), even when controlling for the effect of increasing load on performance (ratio mRT and ratio *d*').

#### Trait individual differences in effort discounting

**Figure 3:**
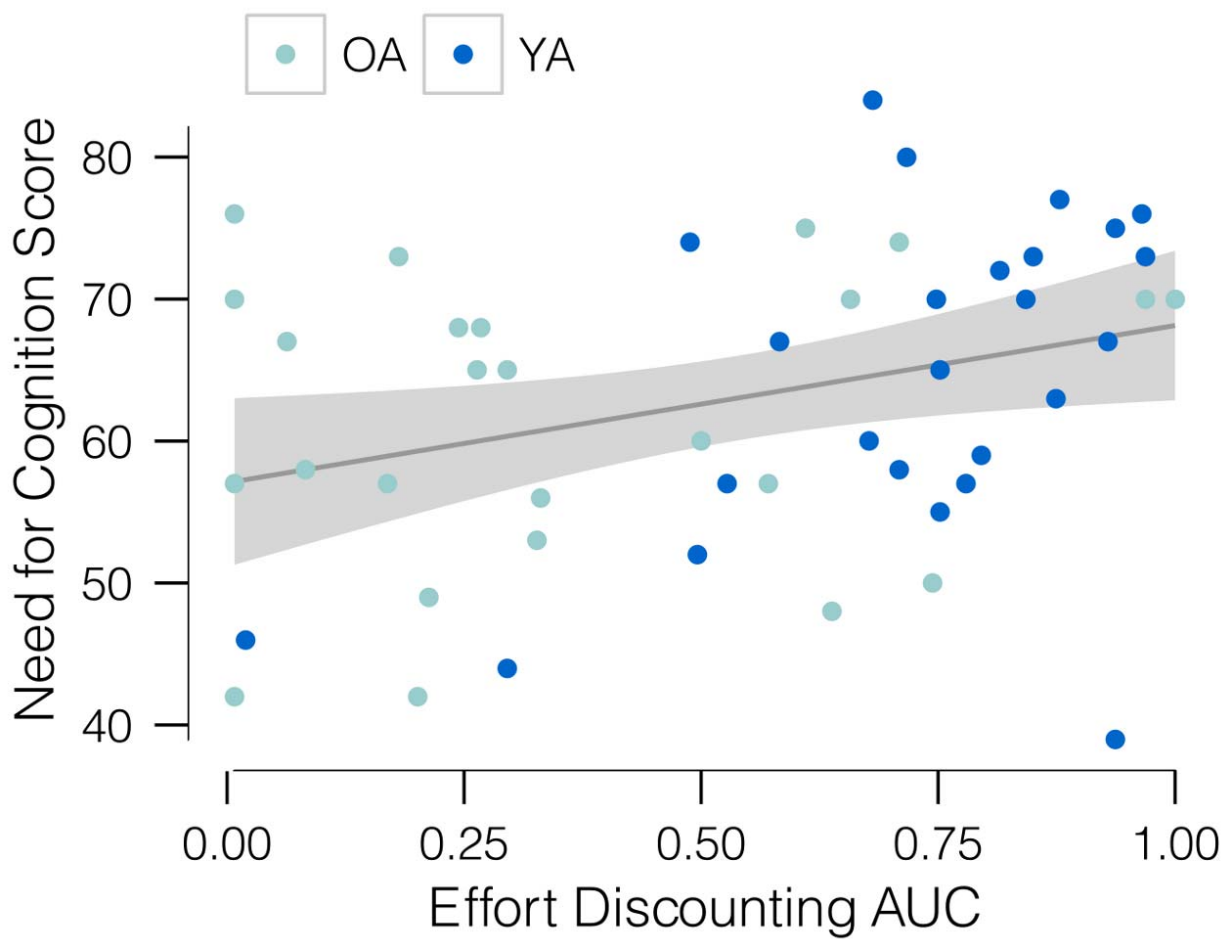
Area under the cognitive effort discounting curve predicts Need for Cognition. Need for Cognition as predicted by Area Under the Curve of subjective value for levels *N* = 2–4 of the *N*-back. *n* = 50 participants.

We next examined individual differences in cognitive effort discounting. For this, we calculated a single area under the curve (AUC_eff.disc._) value for each individual. Area under the curve has been previously used in delay discounting as a desirably atheoretical form of parameter estimation [34]. Trapezoids are bounded by segments connecting SV estimates, thus quantifying effort costliness across *N*-back levels. Levels *N* = 2–4 were used for both YA and OA, so that all AUC_eff.disc._ values were calculated on the same basis. In the sample, AUC_eff.disc._ ranged from 0.015 (easier level always selected during discounting) to 1.0 (harder levels always selected). Thus, both YA and OA varied widely in cognitive effort discounting, ranging from maximum to minimum AUC_eff.disc._ (Figure 3).

We next tested whether discounting is trait-like, predicting willingness to expend cognitive effort in domains other than *N*-back tasks. For this we used the Need for Cognition Scale (NCS), a well-established trait measure of daily engagement with and enjoyment of cognitively demanding activities [14]. A significant positive correlation was observed (Figure 3) between AUC_eff.disc._ and NCS (*B* = 11.07, SE = 4.71, *P* = 0.02, *R^2^* = 0.10), supporting the idea that greater willingness-to-pay to avoid *N*-back effort predicts reduced trait engagement with cognitively demanding activities.

#### Effort discounting detects trait NCS differences better than NTLX self-report

We compared our effort discounting measure to a well-established self-report instrument measuring subjective effort: the NTLX. As with SV, an area under the curve was calculated for each NTLX scale and averaged to create a single NTLX composite score for each individual. This composite and AUC_eff.disc_ were entered into a multiple regression to predict NCS. AUC_eff.disc._ remained as a reliable predictor of NCS (*P* = 0.03), while the composite NTLX score did not (*P* = 0.16). This supports the idea that the objective, behavioral economic preference measured by effort discounting is a better predictor of individual differences in Need for Cognition than are self-report measures of subjective effort.

#### Age differences in effort discounting

Our results strongly suggest that cognitive effort is more subjectively costly for OA than for YA. For each level of the task (*N* = 2–4; Figure 2), average SV was lower for OA than YA (all t-test *P*'s<0.01). Also, AUC_eff.disc._ was reliably larger for YA (Mean_YA_ = 0.72) than OA (Mean_OA_ = 0.36; Wilcoxon rank sum *P*<0.01).

A possible confound is that the observed age differences were primarily driven by age differences in load-related task performance. To address this, another multilevel model (using subject-specific intercepts) was fit to test effects of age, load, and their interaction while controlling for performance. Age (subscript *j*) was dummy coded 1 for OA and 0 for YA. As before, subject-specific intercepts are given a normal distribution with mean and variance terms in the second level of the model. Age group-specific variance terms (subscript *k*) were used since heterogeneous variance yielded a better model fit according to superior AIC/BIC and significant likelihood ratio (4.62; *p* = .03).
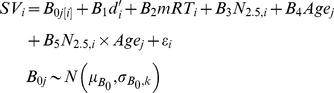
(2)As before, SV decreased with increasing load, and independently with poorer *N*-back performance. Critically, however, after controlling for performance and load, model parameters indicate that: 1) SV is lower for OA than YA, and 2) SV declines with increasing load more rapidly for OA than YA (Table 4).

**Table 4:** Multilevel model of subjective value, as predicted by performance (d'), mean response time (mRT, seconds), and *N*-back level.

Term	*B*	*SE*	*df*	*t*	*p*
(n = 4 levels * 50 participants = 200)					
*d'*	0.07	0.03	146	2.44	0.02
*mRT*	−0.26	0.16	146	−1.68	0.10
*N_2.5_*	−0.11	0.02	146	−4.88	<0.01
*Age*	−0.18	0.06	48	−3.06	<0.01
*Age*×*N_2.5_*	−0.11	0.03	146	−3.80	<0.01

As before, we also fit a model for Equation 2 excluding SV at *N* = 1 (SV_1_). Again, even without SV_1_ in the dataset, we still observed a main effects of load (*B* = −0.17, *SE* = 0.03, *P*<0.01) and the critical effect of age (*B* = −0.31, *SE* = 0.07, *P*<0.01), when controlling for potential age-related performance effects. However, the interaction of age and load, was not significant when SV_1_ was excluded (*P* = 0.90). This latter finding indicates that the interaction reflects age differences in the effect of load specifically from *N* = 1 to *N*>1.

A common approach used to control for between-group differences in subjective effort during task performance is to compare groups in conditions that are matched on performance [35]. Our results clearly show that this method is insufficient. For example, OA performance on *N* = 3 (Mean*_d', OA_* = 1.74) is statistically indistinguishable (t-test *P* = 0.53) from YA performance on *N* = 4 (Mean*_d',YA_* = 1.65), and yet their SV is reliably lower (Wilcoxon *P* = 0.02). Thus, even when matched on performance, OA find cognitive effort subjectively more costly.

#### Effort discounting detects age differences better than NTLX self-report

A comparison of NTLX and COG-ED revealed that our novel paradigm has greater sensitivity to distinguish group-wise subjective experience of cognitive effort. Specifically, the composite NTLX score described above was entered into a logistic regression, along with AUC_eff.disc._, to predict age. AUC_eff.disc._ was a reliable predictor of age (*χ^2^ P*<0.01), while NTLX was not (*χ^2^ P*<0.35), indicating that there is more information about age in effort discounting than NTLX scales.

#### Effort discounting is not merely redundant with Need for Cognition

It is possible that age differences in subjective effort merely reflect age-related variance in NCS since mean NCS scores are numerically (though not reliably *P* = 0.35) lower for OA (Mean_OA_ = 61.60, Mean_YA_ = 64.52). Thus, there is some concern that COG-ED is redundant with Need for Cognition. However, after controlling for NCS scores in a hierarchical regression, age still explained a significant (*P*<0.01) and large (*ΔR* ^2^ = 0.27) increment in variance in AUC_eff.disc._.

### Experiment 2 Results

Replicating a key finding of Experiment 1, there was strong evidence of a linear decline in SV with load. Again, in Experiment 2, reliable linear contrasts obtained for YA, for both the $1 offers (*N* = 2–4, *F*_1,49_ = 13, *P*<0.01, η^2^ = 0.21) and $5 offers (*F*_1,49_ = 15, *P*<0.01, η^2^ = 0.24), and also for OA, for both the $1 offers (*N* = 2–4, *F*_1,46_ = 13, *P*<0.01, η^2^ = 0.22) and $5 offers (*N* = 2–4, *F*_1,46_ = 16, *P*<0.01, η^2^ = 0.26), indicating that effort costs increased with objective load.

Canonical delay discounting behavior includes an amount effect: larger offers are discounted less than smaller offers [36]. Consistent with extant literature, both YAs and OAs discounted hypothetical, delayed offers of $25,000 less than offers of $1,000 (Wilcoxon tests of delay-discounting AUC: *P_YA_* = 0.01, *P_OA_*<0.01). Strikingly, a similar amount effect was also found for cognitive effort discounting: OA AUC_eff.disc._ was reliably (*P* = 0.01) higher for a $5 offer (Mean_$5_ = 0.51) than a $1 offer (Mean_$1_ = 0.43), while YA AUC_eff.disc._ was numerically higher (Mean_$5_ = 0.80, Mean_$1_ = 0.77), though not reliably (*P* = 0.24), likely due to ceiling effects. Also, average subjective value was numerically higher at every load level, for both groups, when the base offer was larger. Thus, for both cognitive effort and delays, participants discounted larger offers to a lesser extent. Our analysis is based on proportional comparisons between amounts (i.e., discounted amount/max amount) not because we assume any particular effort discounting function (indeed there is far too little data to infer a function at this time), but because discounting was clearly not subtractive. The mean discounted value of a $5 offer for *N* = 3, for example, was $3.07 ($1.93 decrement) while the mean discounted value of a $1 offer was $0.60 ($0.40 decrement).

**Figure 4:**
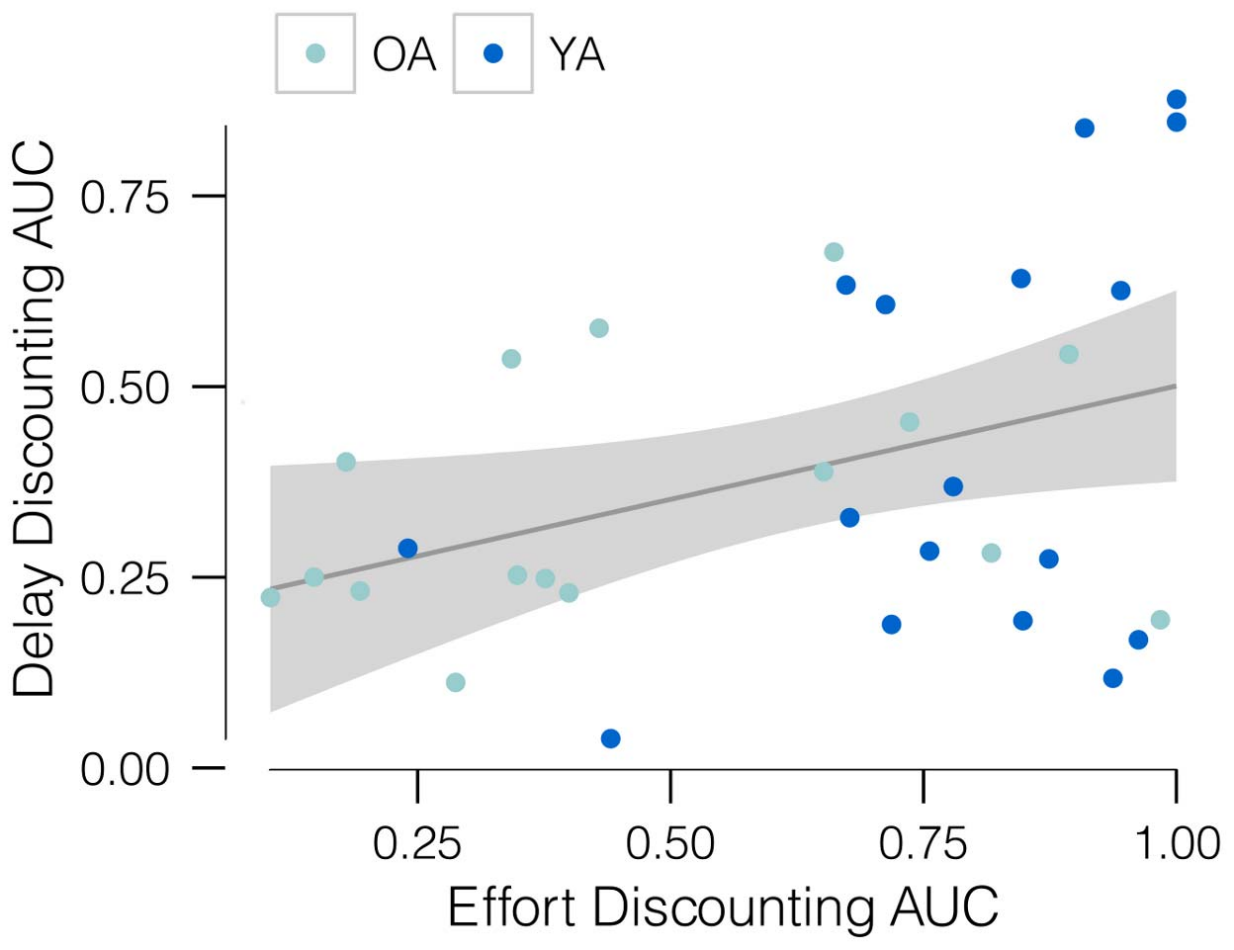
Area under the cognitive effort discounting curve predicts delay discounting. Area under the curves: for effort, averaged across $1 and $5 offers, levels *N* = 2–4; for delay, averaged across $1,000 and $25,000 offers, delays from one week to ten years. *n* = 33 participants.

Delay discounting has been conceptualized as indexing a trait measure of the capacity for self-control and goal-directed behavior [37]. Relatedly, it has been proposed that reduced self-control may relate in part to a bias against cognitive effort (specifically cognitive control demands) [38]. Thus we predicted that those who show greater effort discounting would also show greater delay discounting. Indeed, a positive correlation (Figure 4) was observed between these two discounting domains, supporting the prediction (*B* = 0.30, SE = 0.13, *P* = 0.03, *R^2^* = 0.14).

#### Differences in the marginal utility of money do not explain differences in effort discounting

Delay discounting is sensitive to individual differences in the marginal utility of money. In fact, prior reports that OA show lesser delay discounting [31] were countered by evidence that the effect was primarily due to age-related income differences [39]. Hence, we tested whether age differences in effort discounting are merely the consequence of OA exhibiting lower marginal utility for the offers. In fact, multiple lines of evidence indicate that the observed age effects were not merely an artifact of marginal utility. First, amount effects among OA show clearly that they cared about the utility of our offers. Second, participants rated the extent to which their “choices [were] based on the offer amount ($) of each task.” Consistent with the marginal utility account, OA's average ratings (Mean_OA_ = 5.8) were reliably (*P* = 0.01) lower than YA's (Mean_YA_ = 7.7). However, age (*B* = −0.17; SE = 0.08; *P* = 0.03), but not ratings (*B = *0.01; SE = 0.02; *P* = 0.62), was reliable in a multiple regression of AUC_eff.disc_ (age dummy coded 1 for OA, 0 for YA). Finally, income explained age differences in delay but not effort discounting. Replicating prior reports, a multiple regression of delay discounting AUC showed a main effect of income (*B* = 0.05, SE = 0.02, *P*<0.01), but not age (*B = *−0.08, SE = 0.08, *P* = 0.32). Conversely, a multiple regression of effort discounting AUC showed a main effect of age (*B* = −0.30, SE = 0.09, *P*<0.01) but not income (*B* = 0.02, SE = 0.02, *P* = 0.43). Formally, in a model predicting AUC with age, domain (dummy coded 1 for effort, 0 for delay), and their interaction, the age×domain interaction was statistically significant (*B* = −0.22, SE = 0.11, *P* = 0.05) when income was included as a covariate, but not when income was excluded from the regression model (*B* = 0.13, SE = 0.15, *P* = 0.39). This dissociation suggests that while marginal utility might account for age effects in delay discounting, it does not in COG-ED.

#### Increased Conscientiousness among OA does not explain age differences in effort discounting

Finally, it is possible that OA discount more because they tend to be more conscientious [40] and thus find it more aversive to experience poor task performance. To test whether this hypothesis could explain our age effects, we examined participants' ratings on a scale of 1 (not at all) to 10 (a lot), the extent to which their decisions were “based on whether or not [they] would be able to get a high score.” OA gave numerically, (if not reliably *P* = 0.08) higher ratings (Mean*_OA_* = 6.6) than YA (Mean*_YA_* = 4.9). However, even after controlling for ratings (*B* = −0.03; *SE* = 0.01; *P* = 0.02) in a hierarchical regression, age still serves as a trend-level predictor of AUC_eff.disc._ (*B* = −0.13; *SE* = 0.07; *P* = 0.059). Note that in the follow-up, participants discounted both $1 and $5, so the dependent variable was each participant's average AUC_eff.disc._ across the two amounts.

## Discussion

In this study, we used a novel discounting paradigm (COG-ED) to measure the subjective value of cognitive effort. Our results indicate that people are cognitive misers: they are willing to forgo substantial reward to conserve cognitive effort. Our work converges with the recent studies of Botvinick and colleagues [3], [5], [21], who have shown that individuals are biased to avoid cognitive demand. Importantly, their studies provided one of the first direct links (controlling for task duration and error rate) between task avoidance and demands for cognitive control: they observed that participants reliably avoided conditions involving frequent task switching. Our results replicate and extend this finding in a number of ways. Specifically, we demonstrated that effort costs increase parametrically with increasing load in the *N*-back, a benchmark cognitive control task. Other comparative advantages of the COG-ED paradigm include methodological control of time-on-task and a continuous measure of the subjective effort costs, rather than binary task avoidance.

Botvinick and colleagues have also been interested in examining the economic value of cognitively effortful tasks, conducting a preliminary investigation of this question through post-task self-report. Specifically, [21] asked participants to informally assign a “fair pay” dollar value for completion of task-switching tasks. Note that the values participants assigned (means of $1.89 for more frequent or $0.89 for less frequent task-switching options) for their performance coincided with the range of offer values in our discounting paradigm. While the coincidence of valuations is interesting, asking participants to freely assign values does not confer the principled benefits of revealed preference obtained by a formal discounting procedure. Participant reports that their effort is worth $1.89 are not the same as showing that they are willing to trade their effort for that amount.

Our study also demonstrates the utility of framing the decision to engage in cognitively demanding activities as an economic choice. Subjective value provides an escape from a traditionally circular logic about effort whereby decrements in performance or increased physiological response at increased demands indicate greater effort because they occur at increased demands. Instead, subjective value productively reframes the outcome in precisely those terms of greatest interest to researchers: the extent to which effort costs diminish the rewards of cognitive task engagement. Furthermore, our operationalization places cognitive effort into a well-established discounting framework, illustrating its continuity with a diverse array of subjective cost factors.

We showed that these conceptual advantages translate into several methodological advantages. First, the economic indicator (subjective value) corresponded well with traditional objective (response times and error rates) and subjective indicators (self-report measures) of cognitive effort. Second, it was sensitive to multiple between- and within-subject factors that should impact willingness to expend effort. Within-subject factors include performance and load, and also reward amount. Between subjects, lesser effort discounting predicted greater self-reported engagement with cognitively demanding activities using the NCS. This correlation supports: 1) a trait-like property of effort costliness (some individuals have a higher price point for cognitive effort than others) and 2) that our measure captures that trait. Furthermore, we found an intriguing relationship whereby lesser effort discounting was associated with shallower delay discounting – suggesting that self-control may depend on how costly one finds cognitive effort. Our measures also provide direct support for the claim that cognitive effort is more costly for OA. Importantly, these age differences remain even when controlling for potential confounds like poorer performance, or the reduced marginal utility of money among OA (unlike delay discounting, age differences are not due to differences in income).

Why might OA find cognitive effort more costly? Age-related deficits in dopaminergic function during reward anticipation [41], [42] may play a role. Influential computational models [43] and human and animal work [20], [44], [45] postulate that the invigoration of motor behavior is regulated by the perceived average rate of reward and corresponding dopamine release. If these principles extend to cognitive behavior, OA will be generally less willing to expend cognitive effort as a result of reduced dopaminergic function. Diminished executive function among OA [46]–[48] may also play a role, given the accumulating evidence for a link between cognitive control demands and subjective cognitive effort. In the face of declining capacity, OA may compensate by recruiting a greater fraction of their neural/cognitive control resources [35], [48]; this greater recruitment could underlie the increase in subjective effort. Greater subjective effort may also relate to greater autonomic arousal for OA, when engaged with cognitively demanding tasks [11]. Such autonomic effects could be experienced as particularly aversive for OA [49].

It is impossible to rule out that OA show greater discounting because they value money less than YA. Our approach of manipulating and/or controlling for likely covariates (e.g., reward amount, income, subjective ratings of reward utility) may provide greater confidence that inter-group discounting comparisons are meaningful. However, it is important to note that diminished motivation is deeply confounded with diminished reward sensitivity. Thus, special care should be taken when comparing groups with differential reward sensitivity to make appropriate inferences about subjective effort.

Another limitation is that the elicitation procedure used here never allowed participants to choose to do a harder task for less money. It is possible that some participants may, for example, prefer *N* = 2 to *N* = 1 because they find *N* = 1 relatively boring and would thus prefer to perform *N* = 2 even when offered less money than *N* = 1, if given the option. One solution is to offer equal amounts for high and low load options at the outset, and use participants' first choice to determine which offer is adjusted on the next decision. In this way, indifference points could be estimated, even when they involve lower offers for higher loads.

Our results demonstrate the enormous potential of the discounting framework to investigate both what makes cognitive effort subjectively costly, and also its impact on decision-making and behavior. Future directions could include using effort discounting to explore the extent to which motivation explains apparent cognitive deficits in older adults, or among those with disorders of anergia and avolition, including depression (taking into account concerns about reward sensitivity, as noted above). Effort discounting may also help with interpreting physiological measures thought to relate to cognitive effort. For example, effort discounting could be used to test motivational accounts of age-related discrepancies in neural recruitment and performance in demanding tasks. Moreover, the discounting approach has been productively applied to elucidate the neural mechanisms of delay, risk [16], [17], [20], and physical effort-based decision making [19]. Thus, such methods will likely prove equally valuable for testing formal and neuroeconomic models of cognitive effort [21], [50].
